# The degradation of levofloxacin in infusions exposed to daylight with an identification of a degradation product with HPLC-MS

**DOI:** 10.1038/s41598-019-40201-9

**Published:** 2019-03-06

**Authors:** Andrzej Czyrski, Katarzyna Anusiak, Artur Teżyk

**Affiliations:** 1 The Department of Physical Pharmacy and Pharmacokinetics, Poznań University of Medical Sciences, Święcickiego 6 Street, 60-781 Poznań, Poland; 2 The Department of Forensic Medicine, Poznań University of Medical Sciences, Święcickiego 6 Street, 60-781 Poznań, Poland

## Abstract

In this paper the decomposition product of levofloxacin was identified. Levofloxacin was dissolved in 0.9% NaCl, 5% glucose, and Ringer’s solution. The solutions were divided into two batches: the first one was exposed to daylight and the second one was protected from it. The solutions were stored at the room temperature. The qualitative analysis of the degradation product was performed using MS and TOF detectors. The quantitative assay was done by a validated HPLC method. Visual inspection and pH assessment were done. Levofloxacin protected from daylight remained stable in 0.9% NaCl, 5% dextrose, and Ringer’s solution. A slight decomposition of the analyte was observed in the solutions exposed to daylight with the fastest decomposition rate in Ringer’s solution as compared with 0.9% NaCl and 5% dextrose solutions. The degradation product of levofloxacin detected with MS was levofloxacin N-oxide. Levofloxacin solutions should be protected from direct daylight to maintain drug stability. Levofloxacin N-oxide is formed regardless of the solvent used.

## Introduction

Fluoroquinolones are group of the synthetic antibacterial drugs. They have a broad spectrum of bactericidal activity against bacteria such as *S. pneumoniae, P. aeruginosa, and B. fragilis*. The first fluoroquinolone approved by FDA was nalidixic acid. The introduction of the fluorine atom in the 6^th^ position of quinoline ring developed the antibacterial activity significantly. The fluoroquinolones are divided into four generations. Levofloxacin ((S)-9-fluoro-2,3-dihydro-3-methyl-10-(4-methylpiperazin-1yl)-7-oxo-7H-pyrido[1,2,3-de]-1,4-benzoxazine-6-carboxylic acid) is a representative of the third generation of fluoroquinolones. Levofloxacin exhibits a wide spectrum of bactericidal activity including both Gram-positive and Gram-negative organisms. The activity of levofloxacin depends on its concentration in tissues. The desirable concentration is provided with a repeatable dose in a defined time interval. Once prepared in solution for clinical use, levofloxacin must remain stable to provide effective therapy. Decompensation of levofloxacin once in solution, prior to administration, may lead to a lower than anticipated dose and lack of expected bactericidal effect. Due to the increasing resistance of the microorganisms to the treatment it is essential to administer the proper dose of drug without the degradation products. Moreover, due to the presence of the piperazinyl ring, the decomposition products of fluoroquinolones might cause photoallergy^1–3^.

Levofloxacin stability was assessed in different matrices. It was exposed to different stress factors such as UV-light, ozone, and acidic/basic hydrolysis. The decomposition products were detected using MS techniques^4–8^. In our study the decomposition product was detected in the MS analysis supported with GC-TOF. The matrices used in our study were the infusions (Ringer’s solution, 0.9% NaCl, and 5% dextrose) exposed and protected from daylight at the room temperature. These solutions are the most common in clinical practice and might be considered as potential solvents for levofloxacin. This study presents the degradation product which might be formed during the use of the drug (in-use stability). We also conducted the forced degradation of levofloxacin in a solar simulator to check if there were any differences in the formed decomposition products. Our manuscript comprises both the application study (in-use stability test) and the base study (the degradation in the solar simulator) concerning levofloxacin degradation in commonly used infusions.

## Results and Discussion

### The development of HPLC-UV method

The qualitative analysis was performed with a validated HPLC method. The mobile phase consisted of acetonitrile and 0.4% triethylamine solution (24:76, v/v) which was an ion pair reagent. This proportion provides the total resolution of the levofloxacin, internal standard (IS) and the degradation product. The addition of an ion pair reagent causes better interaction of levofloxacin with the stationary phase and it reduces the tailing of the peaks when combined with the proper value of pH. According to the literature, use of an ion pair reagent up to a concentration of 1% combined with a slightly acidic pH provides good resolution of the analytes^9^. The silanol groups are ionized at the pH above 3.5 and they interact with 1° and 2° amines^10^. The pH of the mobile phase was 2.5. The increase of pH causes the peak tailing. The separation mechanism is based on an interaction of the analyte with the free silanol groups of the stationary phase. The other factor that was considered for improving the shape of the peak was the use of a phosphate buffer, however the use of a such did not improve the quality of the resolution of analytes significantly. The applied organic solvent was acetonitrile. The use of methanol resulted in a longer time of analysis. The peaks were broad and the pressure on the column was high, the resolution was poor. The mixtures of acetonitrile and methanol in different proportions were also tested but it resulted in poor resolution.

The intraday CV and RE were within the range 1.51–1.70% and 0.22–1.54%, respectively (Table 1). The interday CV and RE were 2.77–4.36% and 0.74–4.07%, respectively. They obey the ICH recommendations for validation of analytical methods. The method was linear – it was proved by the Mandel’s test where test value was 0.4667 vs. table value 34.11. The value of the intercept was not statistically significant, and it was omitted. The recovery exceeded 99%.

**Table 1:** The validation parameters.

Nominal concentration of levofloxacin	Inter-day assay (n = 5)		Intra-day assay (n = 5)	
[mg/mL]	CV^a^	RE^b^	CV^a^	RE^b^
2.0	3.58	4.07	1.51	0.71
3.0	4.36	1.20	1.70	0.22
5.0	2.77	0.74	1.67	1.54

^a^CV – coefficient of variation, ^b^RE- relative error.

The analyte remained stable in stability tests. The RE did not exceed 2.0% after both storage for 24 hours at room temperature and storage at −20 °C for three months. After three freeze-thaw cycles the RE did not exceed 2.2% (Table 2).

**Table 2:** The stability of levofloxacin in the samples.

Experimental conditions	Concentration, mg/mL		
	2.0	3.0	5.0
*After storage at room temperature for 24 h*			
Mean assayed value, mg/mL	2.01	0.98	5.10
Accuracy, %RE	0.5	2.0	2.0
*After three freeze-thaw cycles*			
Mean assayed value, mg/mL	2.04	1.01	5.07
Accuracy, %RE	2.2	1.0	1.4
*After storage at −20 °C for three months*			
Mean assayed value, mg/mL	2.03	1.02	5.08
Accuracy, %RE	1.5	2.0	1.6

**Figure 1:**
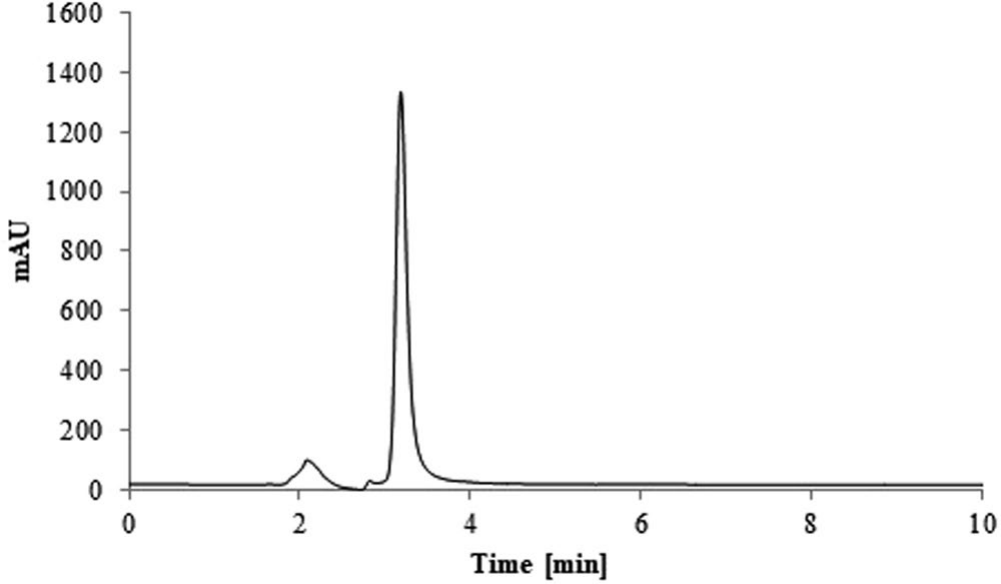
The chromatogram of the HPLC analysis for the levofloxacin solution (5 mg/mL) (levofloxacin t_r_ – 3.17 min).

**Figure 2:**
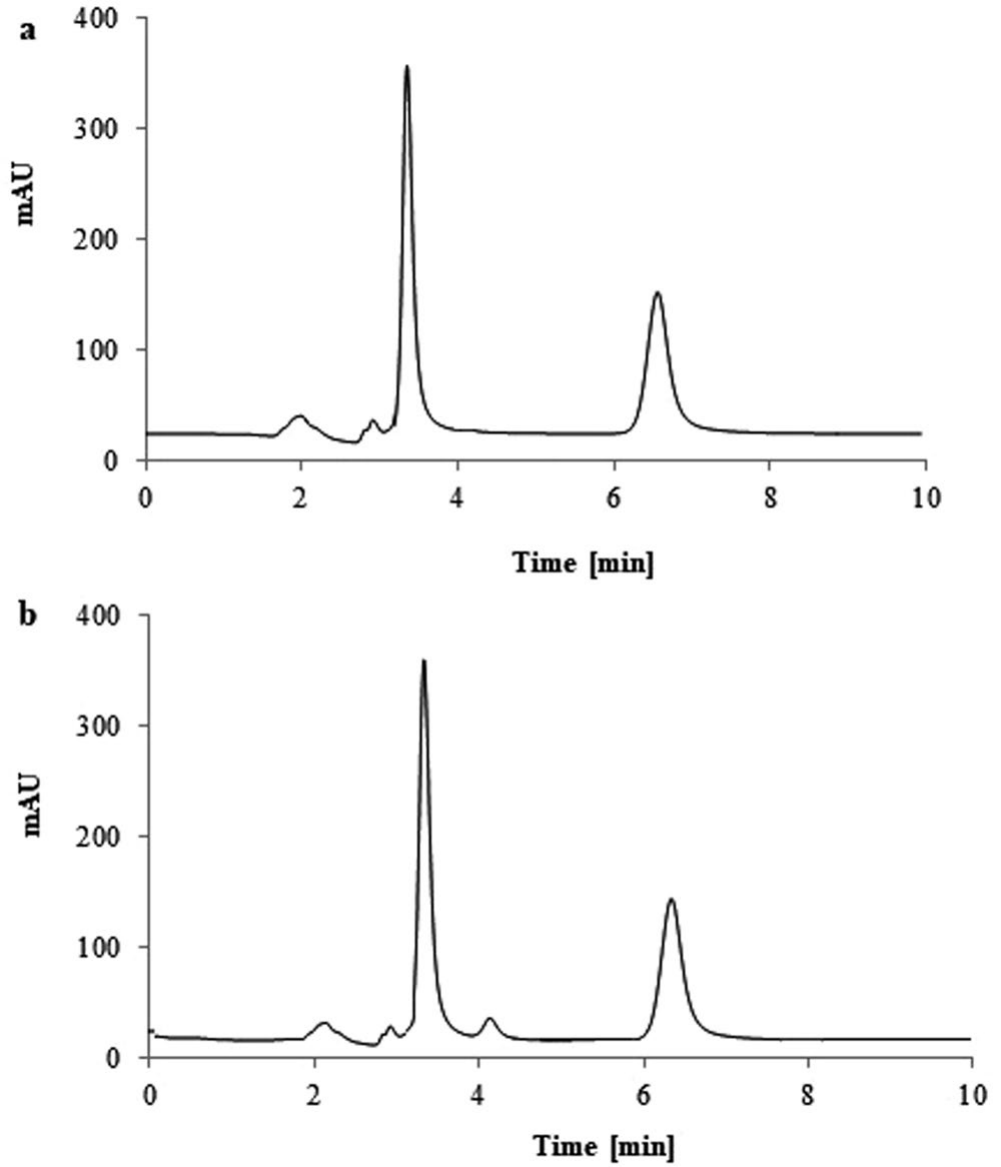
The chromatogram of levofloxacin: (**a**) dissolved in Ringer’s solution at day 0^th^ (time-zero analysis), (**b**) dissolved in the Ringer’s solution exposed to daylight at 84^th^ day (levofloxacin t_r_ – 3.10 min., levofloxacin N-Oxide t_r_ – 4.05 min., IS – 6.56 min).

The chromatogram of the solution of levofloxacin is presented in the Fig. 1. There were no additional peaks that could interfere with an IS peak (Fig. 2). The chromatogram of the investigated solution is shown in a Fig. 2b. All the peaks were fully resolved. The decomposition product peak was detected in addition to the levofloxacin and IS peaks. The appearance of the additional peak was associated with the change in the color of the solutions. The color was intact for the solutions protected from any source of light. The color of the solutions exposed to daylight and solutions exposed to lamp light in a solar simulator became more intense, which implied that decomposition of levofloxacin took place.

### The LC-MS analysis

**Figure 3:**
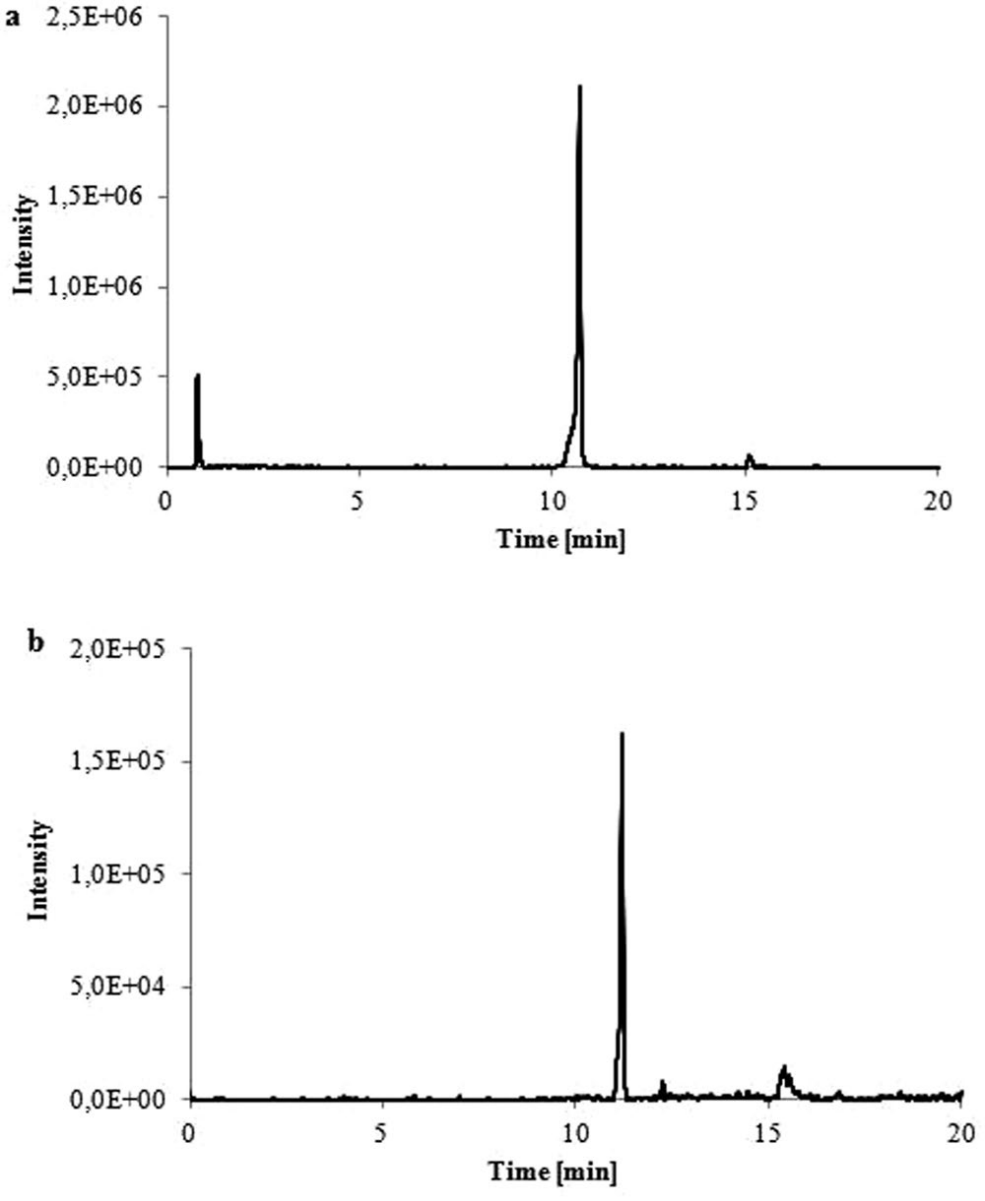
The LC-MS chromatograms of the following ions: (**a**) levofloxacin m/z 362 (t_r_ – 10.7 min); (**b**) levofloxacin N-oxide m/z 378 (t_r_ – 11.2 min.).

**Figure 4:**
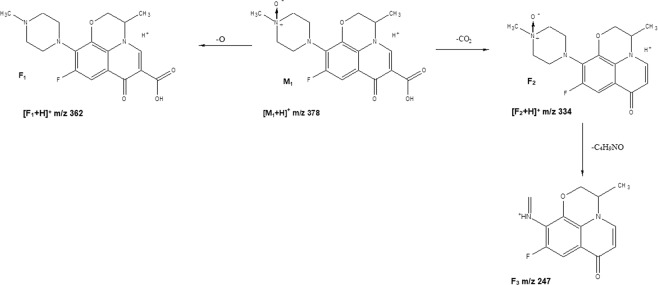
The fragmentation pathways of levofloxacin N-oxide (M_1_- levofloxacin N-oxide, F_1,_ F_2,_ F_3_ – fragmentation ions).

The levofloxacin solution samples collected at the beginning of the experiment were injected onto LC-MS system. The LC-MS analysis of levofloxacin resulted in m/z 362 ion (Fig. 3). In the LC-MS analysis of the solutions exposed to light the degradation product was detected. It was m/z 378 [M_1_ + H]^+^ (Fig. 4). The GC-MS with TOF detection also confirmed the presence of the degradation product, a compound with molecular weight 377. This implies the presence of the additional oxygen in the molecule. The new compound could be either the N-oxide or hydroxy derivative. Ge *et al*.^11^ reported the presence of a degradation product with a molecular weight 377. It was a result of the hydroxylation in the piperazine ring. The fragmentation ions in our study that were observed are m/z 362 (F_1_ + H^+^) which was due to removal of an oxygen atom from the levofloxacin N-oxide molecule and m/z 334 (F_2_ + H^+^) which was a result of decarboxylation of the ion m/z 378 (M_1_) (Fig. 4). Tong *et al*.^12^ observed the removal of an oxygen atom in the N-oxides during MS analysis. It is characteristic for N-oxides fragmentation and makes it possible to distinguish them from the hydroxylated compounds. In the MS spectrum the ion m/z 247 [F_3_] was also detected. It was a result of the removal C_4_H_9_NO from the ion m/z 334. The analogic fragmentation was observed by Tang *et al*. for fluoroquionolones with piperazine moiety^13^. The ions m/z 344 and 318 were also observed. They were the result of the removal of water and CO_2_ from the ion m/z 362 [F_1_ + H]^+^. The loss of water was not observed for the m/z 378 ion. According to Ramanathan *et al*.^14^ the loss of water in N-oxides during ESI-MS fragmentation is not significant. The opposite is observed in aliphatic hydoxylated compounds where the lost of water is predominant. It also confirmed the presence of N-oxide. The neutral loss during the fragmentation process is characteristic for the fragmentation of fluoroquinolones^13,15^. The degradation product of levofloxacin in the investigated compounds was levofloxacin N-Oxide.

The presence of the ion (m/z 378) was observed by Wang *et al*.^16^, Lalitha Devi *et al*.^17^ and De Witte *et al*.^4^. N-oxide is formed due to the presence of electrons from the methyl group, which is a better donor than hydrogen. It results in higher electron density at the tertiary nitrogen atom at the piperazine ring^4^. Ciprofloxacin, which possesses secondary amine nitrogen atom at the piperazine ring, does not form N-oxide^18^. Sturrini *et al*.^19^ exposed levofloxacin to solar simulator dissolved in wastewater and several degradation products were detected. The degradation products arose from the removal of the methyl group from piperazine ring, the substitution of fluorine atom with hydroxyl group, or dehalogenation. The degradation products were detected not only in wastewater^19^ but also in untreated river water^7^. Ge *et al*.^12^ proposed main transformation pathway for hydroxyl radical photooxidation for levofloxacin in pure water. The primary degradation products were the result of hydroxylated defluorination or hydroxylation and removal of the piperazinyl ring. Ge *et al*.^20^ found also that photoinduced decarboxylation, defluorination, and also piperazinyl dealkilation depend on the structure of the analysed fluoroquinolone.

According to the literature, levofloxacin N-oxide does not possess any bactericidal activity^21^. Its presence as a degradation product may lead to the lack of the bactericidal effect and increase of bacterial resistance to the active agent. Levofloxacin N-oxide might also be a potential mutagen due to the presence of a quinolone-3-carboxylic acid or naphthyridine analog. Zhu *et al*. confirmed that levofloxacin could not be considered as a genotoxic impurity^22^.

### The stability study

Levofloxacin remained stable in the solutions protected from daylight. For solutions exposed to daylight the opposite is observed as a decrease in levofloxacin drug level was found. The most significant decrease was observed in Ringer’s solution. At the 84^th^ day the observed concentration in Ringer’s solution was an approximately 85% of the initial concentration of levofloxacin.

At the 84^th^ day the observed concentration of levofloxacin exposed to daylight formulated in 5% dextrose and 0.9% NaCl was approximately 96% of the initial concentration (Table 3). The values of the rate constant were: 1.99 × 10^−3^ [day^−1^], 7.48 × 10^−4^ [day^−1^] and 5.11 × 10^−4^ [day^−1^] for levofloxacin dissolved in Ringer’s solution, 0.9% NaCl, and 5% dextrose, respectively. The stability estimated from Equation1 (‘The constant rate calculation’ section) shows that 10% of levofloxacin is decomposed within 53 days in Ringer’s solution, 141 days in 0.9% NaCl and 206 days in 5% dextrose for the solutions exposed to daylight. The lower stability of levofloxacin in the Ringer’s solution exposed to daylight may be associated with the presence of calcium ions as the fluoroquinolones have an affinity for divalent ions. They form complexes which are unstable, and it may affect the stability of the drugs^23^.

**Table 3:** The changes of the concentration of levofloxacin in the solutions exposed to and protected from daylight.

Solution	Solutions Exposed To Daylight							
	Initial concentration [mg/mL]	Day of storage						
		7	14	21	28	42	56	84
		% Initial concentration remaining						
0.9% NaCl	5.07	100.77	100.18	99.10	98.87	97.90	96.61	95.11
5% Glucose	5.07	100.02	99.24	98.72	98.32	97.80	96.91	96.01
Ringer’s Solution	5.04	100.80	97.45	95.89	93.06	90.44	88.77	85.60
	**Solutions Protected From Daylight**							
0.9% NaCl	5.05	101.89	101.56	100.91	100.49	101.20	100.98	99.11
5% Glucose	5.07	100.00	99.63	99.21	99.23	98.28	98.40	99.80
Ringer’s Solution	5.07	101.69	100.137	99.21	99.25	99.48	98.55	98.41

The degree of degradation of levofloxacin in the samples exposed to the solar simulator was similar to the samples exposed to daylight. Samples exposed to the solar simulator in Ringer’s solution, 0.9% NaCl, and 5% dextrose yielded a levofloxacin concentration of 88%, 95%, and 92% of the initial concentration, respectively. The measurements conducted in the solar simulator confirmed the results from the in-use stablility test - levofloxacin decomposed the fastest in the Ringer’s solution and the slowest in 0.9% NaCl solution when exposed to the light. The samples protected from the light in the solar simulator remained stable.

The stability of levofloxacin in 5% dextrose, 0.9% sodium chloride, and Ringer’s solution protected from daylight was confirmed by the Williams *et al*.^8^ and van den Bussche *et al*.^24^. Williams *et al*. conducted the study in polyvinyl chloride bags. In our study the Ringer’s solution sample was in a polyethylene bag. The 0.9% NaCl and 5% glucose samples were in a polyolefin/polyamide bag. Levofloxacin N-oxide is formed in the all solutions exposed to daylight regardless the material used. The rate of levofloxacin decomposition in the solutions exposed to daylight was slow. If there is no possibility to protect the solution of levofloxacin, it should be used *ex tempore*.

## Conclusions

Levofloxacin dissolved in Ringer’s solution, 0.9% NaCl, and 5% dextrose remained stable in the solutions protected from daylight. Levofloxacin decomposes in the solutions exposed to daylight but the decomposition is not rapid. The shortest t_0.1_ measured was in Ringer’s solution. The levofloxacin degradation product identified was inactive levofloxacin N-oxide. It is formed regardless of the used solvent. Solutions of levofloxacin should be protected from daylight to maintain drug stability or otherwise used immediately.

## Material and Methods

### Standards and reagents

Levofloxacin and triethylamine were obtained from Sigma-Aldrich Chemie (Steinheim, Germany). Moxifloxacin was purchased at SantaCruz Biotechnology (USA). Acetonitrile, methanol was purchased from Merck (Darmstadt, Germany) and 85% orthophosphoric acid from Fluka (Germany). All the reagents were of the HPLC purity grade. Formic acid (Fluka, Germany) for LC-MS was used. Ultra-pure water was used. 0.9% NaCl (Lot 13J27G61), 5% dextrose (Lot 14G18E1K), and the Ringer’s solution (Lot 1310213) were purchased at Baxter.

### Calibration standards

The concentration of levofloxacin solution was 12 mg/mL and moxifloxacin (IS – internal standard) 1 mg/mL. The working solutions of levofloxacin were prepared by the dilution of the stock solution. The concentration range was 1–6 mg/mL. The concentration of the working solution of IS was 150 mg/L. The linear regression equation describing the relationship between levofloxacin concentration and the peak area was determined by the least squares method. 10 μL of the analysed solution was diluted with 990 µL of a proper solvent (0.9% NaCl, 5% dextrose or Ringer’s solution). 100 μL of the dilution was mixed with 20 μL of the working solution of IS and filled up to 200 μL. The dilution was injected onto HPLC system. The volume of an injection was 20 µL.

### The investigated solutions

Levofloxacin was dissolved in a proper solvent (0.9% NaCl, 5% dextrose, or Ringer’s solution) under aseptic conditions. The color of the solutions was yellow. The final concentration was 5 mg/mL. The solvents were isotonic – the osmolarity was within the range 290–298 mOsm/kg. The dissolution of levofloxacin in the solvent did not change the osmotic pressure significantly. Visual stability was defined as a retention of the original clear, transparent yellow and visually particulate-free solution. One set for each solvent consisted of 6 bags. Three of them were exposed to daylight, and the remaining three were stored in the dark – they were covered with foil. All the bags were kept inside the building, stored at the room temperature (23.7 °C ± 0.7 °C). The samples were collected in Spring (84 days). 23 days were cloudy, 35 days were partly cloudy and 26 days were sunny.

The solutions of levofloxacin in the investigated infusions were also exposed to solar simulator equipped with the xenon lamp. This lamp meets the D65/ID65 emission standards defined by ISO. The test obeyed the ICH quidelines. The samples protected from the light with an aluminium foil were used as dark controls. The power of the source of light was 250 W/m^2^. The samples were analysed with HPLC-UV method.

### The HPLC-UV analysis

The analytes were detected with UV detector (detection wavelength λ = 295 nm), the temperature was ambient. The flow rate was 1 mL/min. The elution was isocratic. The mobile phase consisted of acetonitrile and 0.4% triethylamine solution adjusted to pH 2.5 with ortophosphoric acid (24:76, v/v). The LiChroCART column (250 × 4 mm, 5 μm, Merck Germany) with LiChroCART guard column (4 × 4 mm, 5 μm, Merck Germany) were applied for chromatographic separation. The method was validated according to ICH (International Council for Harmonisation) guidelines. The peak of the decomposition product was separated from the LEVO peak (R_s_ = 2.9). The chromatograms are shown in Fig. 1 and in Fig. 2. The blank samples injected onto HPLC system did not show any peaks.

#### The validation parameters

The following validation parameters were assayed linearity, precision (CV – coefficient of variation) and accuracy (RE-relative error), specificity, stability and recovery.

The linear equation describes the relationship between LEVO concentration and the area under the peak of LEVO and IS ratio. The calibration curve was calculated by the least squares method. Five calibration curves were prepared on five separate days and the validation parameters were calculated.

Intra-day and inter-day CV and RE were estimated for control samples at the following concentrations: 2.0, 3.0 and 5.0 mg/mL (Table 1).

The stability was evaluated during three freeze-thaw cycles, after storage for three months at the temperature −20 °C and after storage at a room temperature 24 h. The analytes are stable if the deviation from the nominal concentration is within ±10% (Table 2).

The recovery was determined by comparing the levofloxacin/IS area ratios obtained from the infusions spiked with a known amount of levofloxacin solution to the ones of water spiked with levofloxacin solution. The recovery was tested for the following concentrations: 2 mg/mL, 3 mg/mL and 5 mg/mL.

### The MS analysis

#### The LC-MS analysis

10 μl of investigated solution was diluted with methanol to 1 mL, then 20 μl of the above dilution was further diluted with methanol to 1 mL. The mixture was injected onto MS system. LC-MS measurements were performed on a 1200 series liquid chromatograph coupled with a 6410B Triple Quad mass spectrometer (Agilent, USA). Separation was performed at 40 °C with a Poroshell 120 EC-18 column (3.0 × 75 mm, 2.7 µm, Agilent, USA). Mobile phases were: 0.1% formate buffer pH 3.2 [A] and 0.1% formic acid in acetonitrile [B]. Gradient elution was programmed as follows: 95% [A] and 5% [B] for 5 min., followed by a linear change of 80% [A] and 20% [B] in 3 min., then linear change to 5% [A] and 95% [B] in 5 min. and was held for 7 min., then by a linear change of 95% [A] and 5% [B] for 2 min. Post time was 2 min. and total chromatographic cycle was 24 min. The flow rate was 0.5 mL/min. The instrument was operated with electrospray ionization (ESI) source in the positive mode. MS conditions were: drying gas temperature (nitrogen), 300 °C; nebulizing gas flow, 8 L/min; nebulizing gas pressure, 40 psi; capillary voltage, 4 kV; fragmentor voltage, 50–250 V. The acquisition was carried out in the scan mode (m/z 50–650).

#### The GC-MS-TOF analysis

GC–MS measurements were performed on an Agilent 7890 gas chromatograph (Agilent USA) with an L-PAL3 autosampler (Leco USA) coupled with a Pegasus BT TOF (time of flight) mass spectrometer (Leco, USA). Separation was performed on HP-5 MS column (30 m × 0,25 mm, 0,25 µm, Agilent, USA). Injection 1 μl splitless, injector temperature 250 °C. Oven program was as follows: 50 °C (1 min.) to 300 °C (10 °C/min.) 5 min. The carrier gas was helium, constant flow 1 mL/min. Transfer line temperature was 250 °C. Mass spectrometer parameters was as follows: source temperature 250 °C, mass range 40–650 m/z and acquisition rate 20 spectra/s.

### The constant rate calculation

According to the literature data, levofloxacin’s decomposition obeys the first-order kinetics^25^. The changes in the concentration of levofloxacin are given in a Table 3. The constant rate (slope of the curve) was calculated according to the first-order kinetics linear equation. The time when 10% of the drug decomposes was calculated by the Equation 1:

1 $${{\rm{t}}}_{0.1}=(0.1054/\text{slope})$$

The samples for analysis were collected at the following days - 0, 7, 14, 21, 28, 42, 56 and 84.
